# Dr. G. W. Parmly

**Published:** 1852-01

**Authors:** 


*Dr. G. W. Parmly.*—We have recently had the pleasure of a visit from Dr. G. W. Parmly, dentist to the Royal Family at the Hague, and son of Dr. L. S. Parmly, of New Orleans. After an absence of six years, he has returned to visit his friends in the United States, and although he still holds his appointment abroad, we trust he may be induced to resign it, and resume his practice in New Orleans. We do not like to part with so good a dentist, even though it be to minister to the wants of royalty. We trust that ere long, Dr. P. will favor us with an opportunity of giving our readers the result of his observations on the state of the dental profession in Europe.

